# A Renewed Vision for Biological Cybernetics

**DOI:** 10.1007/s00422-020-00837-7

**Published:** 2020-06-26

**Authors:** Lindner Benjamin, Peter J. Thomas, Jean-Marc Fellous

**Affiliations:** 1 Physics Department, Humboldt University Berlin, Newtonstr. 15, 12489 Berlin, Germany; 2 Bernstein Center for Computational Neuroscience Berlin, Philippstr. 13, Haus 2, 10115 Berlin, Germany; 3 Department of Mathematics, Applied Mathematics, and Statistics, Case Western Reserve University, 10900 Euclid Avenue, Cleveland, OH 44074 USA; 4 Departments of Psychology and Biomedical Engineering, University of Arizona, 1503 E. University bld, Tucson, AZ 85721 USA

With this edition of *Biological Cybernetics* we implement several changes in the Journal. Attentive readers may have already noticed the differences in the Journal’s aims and scope, the cover page, and, most obviously, the subtitle, which now reads

*Advances in Computational Neuroscience and in Control and Information Theory for Biological Systems*

Of course, computational neuroscience (subject of the previous subtitle) remains a central field of application of Cybernetical principles in biology, and we do not intend to weaken the Journal's impact in this field. Instead, we aim at widening the scope to biological systems beyond neuroscience where principles of control and information theory are finding more and more fruitful utilization.

At one end of the spectrum, for example at the single-cell level, there are many forms of feedback and information processing in the biochemical cascades triggered at membrane receptors to which the tools that have been successfully applied to Neuroscience can be applied, including mathematical and theoretical tools of information theory, control theory and the theory of stochastic processes. Thus the growing fields of systems biology and synthetic biology fall also within the scope of *Biological Cybernetics.* At the other end of the spectrum, for example when studying the collective information processing in flocks and herds of animals and collective forms of decision making in humans, Cybernetical principles are certainly at work as well. As such, in the future, we will also consider studies of such systems (which are not in the strict sense subjects within Computational Neuroscience) for publication in *Biological Cybernetics*.

To place this broadening of scope in historical context, we are reminded that at its founding in 1961, *Biological Cybernetics* described itself as “A Journal Dealing with the Transmission and Processing of Information as well as with Control Processes in both Animals and Automata.” The original aims and scope have never been stated explicitly in an editorial, and cannot be found in the journal’s digital archives. However, from 1961 to 1968 they were printed on the back cover and may still be found in libraries that have not yet destroyed their hard copy in favor of digital holdings. We reproduce the English version of the original German aims and scope here:

The concepts of transmission of information, processing of information and automatic control engineering originated within technology and physics. Today, however, these concepts have also found application in the biological sciences. The logical procedures, the methods of experimental and theoretical approaches and the mathematical techniques that are applicable to the physical sciences can often be successfully transferred to the realm of the life sciences. By applying those techniques to sensory and neurophysiological problems, new insight has been gained into the principles underlying how organisms handle information. Conversely, physicists and engineers have shown increasing concern for the mechanisms that organisms have evolved in the areas of communication and control. The understanding of these mechanisms may have significant technological consequences as, for instance, in relation to the nature of learning processes.

»Kybernetik« would like to cultivate experimental and theoretical findings in the following fields: Information theory; theory of automata; theory of control systems; mathematical foundations of communication theory; sensory processes and micro- and macrophysiology of the central-nervous-system in relation to information handling; information handling by organisms (incl. man) and task oriented groups; mathematical models for communication and control processes in organisms.

Thus, while aspects of neural systems ranging from sensory integration to motor control provided paradigmatic examples of control and information processing, the original scope of *Biological Cybernetics* was not limited to computational neuroscience per se. Indeed, the most prominent works published in the first two decades of the journal included not only Kohonen’s (1982) self-organizing feature maps, Von der Malsburg’s (1973) theory of orientation column formation, and the Wilson-Cowan equations (1973), but also Gierer and Meinhardt’s (1972) theory for pattern formation during morphogenesis in multicellular organisms.

The Journal's new cover art reflects the renewed extent of its scope. Single cell and brain represent the diversity of subjects of modern Cybernetics; the integral sign stands for the mathematical and quantitative nature of the cybernetic approach, and the feedback arrows remind us of the central role of control principles.

We think that these changes are timely and will open the journal to new audiences, realizing that the general principles of Cybernetics have applications in wide ranging subdisciplines of biology.
